# Energy Intake-Dependent Genetic Associations with Obesity Risk: BDNF Val66Met Polymorphism and Interactions with Dietary Bioactive Compounds

**DOI:** 10.3390/antiox14020170

**Published:** 2025-01-30

**Authors:** Ting Zhang, Sunmin Park

**Affiliations:** 1 Department of Bioconvergence, Hoseo University, Asan 31499, Republic of Korea; zhangting92925@gmail.com; 2 Department of Food and Nutrition, Obesity/Diabetes Research Center, Hoseo University, Asan 31499, Republic of Korea

**Keywords:** obesity, genetic variants, bioactive compounds, BDNF, molecular docking

## Abstract

Obesity represents a complex interplay between genetics, nutrition, and lifestyle. This study aimed to elucidate the intricate relationship between genetic variants, energy intake, and bioactive compounds in influencing obesity risk, particularly in low energy intake, to reveal how dietary intake modulates molecular-level interactions. We analyzed 53,117 participants stratified by obesity status and energy intake levels. Genome-wide association studies explored the genetic variants associated with obesity risk in low-energy- and high-energy-intake subgroups. Advanced computational approaches, including molecular docking, k-means clustering, and uniform manifold approximation and projection (UMAP), were employed to analyze interactions between missense variants and natural compounds. Ten genetic variants were significantly associated with obesity, particularly in participants with low energy intake. The most prominent variants included brain-derived neurotrophic factor (BDNF) Val66Met polymorphism (rs6265). Molecular docking identified 152 bioactive compounds with strong binding affinity to BDNF Val66Met, including 107 compounds binding to both wild and mutant types. Citrus fruits and green vegetables showed selective binding to the mutant type. Antioxidant nutrient intake (anthocyanins, isoflavonoids, vitamins C and E, selenium) was higher in lean versus obese individuals in the high-energy-intake group. Alcohol consumption and selenium intake modulated polygenic risk scores’ influence on obesity risk in high-energy-intake individuals. Notably, citrus fruit intake correlated with lower BMI across all *BDNF* rs6265 genotypes. In conclusion, energy intake-specific genetic associations with obesity and identifies potential bioactive compounds for targeted interventions. The findings suggest that antioxidant nutrient intake, particularly from citrus fruits, may help manage obesity risk, especially in individuals with specific genetic variants.

## 1. Introduction

Obesity is a global public health crisis characterized by an imbalance in energy homeostasis, where energy intake exceeds energy expenditure over an extended period [1]. This disturbance in the delicate balance between caloric intake and caloric utilization leads to the accumulation of excess body fat, which can impact an individual’s long-term health and well-being [1]. However, some individuals develop obesity even when their energy intake is lower than their estimated energy requirement (EER), while others can maintain a healthy weight despite consuming more calories than their EER, suggesting the role of genetic factors [2]. This heterogeneity in the development of obesity highlights the need for a better understanding of the interplay between genetic factors, energy intake patterns, and the role of bioactive nutrients [3]. Previously, animal studies have demonstrated that increased fat deposition is associated with below-normal levels of antioxidants [4,5].

The primary approaches to obesity management have focused on negative energy balance through lifestyle interventions, such as dietary modifications and physical activity [6]. While these strategies can be effective for some individuals, they often fail to produce sustainable weight loss, especially in those with a strong genetic predisposition to obesity. The activation or inhibition of the genes linked to food intake and energy expenditure can promote weight loss through enhanced satiety signaling and delayed gastric emptying [7]. There is a growing interest in the potential of natural bioactive compounds, particularly flavonoids (including anthocyanins), that act on obesity-related genes in obesity prevention and management [4,8,9]. These plant-derived phytochemicals have demonstrated promise concerning their anti-inflammatory and antioxidant effects and role in metabolic regulation that may interact with obesity-associated genetic variants to mitigate the risk of weight gain.

Numerous genome-wide association studies (GWASs) have identified several genetic variants associated with obesity. However, most of these studies have focused on the direct relationship between genetic factors and obesity, primarily using body mass index (BMI), waist circumference, and body fat percentage [10,11]. While these studies have revealed interactions between polygenic risk scores (PRSs) and energy intake in influencing obesity [10,11], there remains a significant gap in understanding the genetic variants specific to obesity and their interaction with energy intake. Understanding how genetic factors interact with nutritional factors, especially bioactive compounds like flavonoids, is crucial to developing more personalized approaches to obesity prevention and management [12,13]. Despite the potential importance of these interactions, few studies have comprehensively explored the genetic variants related to obesity in the context of individual energy intake levels.

To address this gap, this study aimed to investigate the complex interplay between genetic variants, energy intake levels, and bioactive compounds on obesity risk. We aimed to reveal how molecular-level interactions can be modified by dietary intake. Specifically, the study sought to achieve the following: (1) identifying the genetic variants associated with obesity risk in individuals with a lower energy intake than their EER; (2) providing a molecular-level understanding of how bioactive compounds interact with genetic variants; and (3) evaluating the potential of targeted nutritional interventions to modulate genetic obesity risk. The findings of this study have important implications for managing the global obesity epidemic by developing more personalized and effective approaches to obesity prevention and treatment. Ultimately, this knowledge can potentially improve public health outcomes and reduce the burden of obesity-related comorbidities.

## 2. Methods

### 2.1. Participants

This study recruited Korean adults aged over 40 years from a hospital-based urban cohort (n = 58,630) as part of the Korean Genome and Epidemiology Study (KoGES) conducted between 2004 and 2013. Participants with medical histories potentially influencing energy metabolism, including cancers, thyroid diseases, chronic kidney disease, and brain-related diseases, were systematically excluded (n = 5513). The study protocols were approved by the Institutional Review Board of the Korea National Institute of Health (KNIH; KBP-2015-055) and Hoseo University (1041231-150811-HR-034-01), with all participants providing written informed consent.

### 2.2. Demographic, Anthropometric, and Biochemical Data Collection

A trained technician conducted comprehensive interviews to gather detailed demographic information, including age, gender, area of residence, education, income, occupation, alcohol consumption status, smoking status, and exercise habits. Socioeconomic factors were stratified: household income was categorized into very low, low, intermediate, and high levels, while education status was classified as less than high school, high school, or college and above [14].

Participants underwent standardized anthropometric assessments, including measurements of body weight, height, and waist circumference. Body mass index (BMI) was calculated by dividing the weight (kg) by squared height (m^2^). Advanced machine learning techniques were applied to predict skeletal muscle mass and body fat content, utilizing data from the Ansan/Ansung cohort as a training dataset [15,16]. Skeletal muscle index (SMI) was determined by dividing the skeletal muscle mass measured with an Inbody body composition analyzer (Seoul, Republic of Korea) by height (m^2^). Blood pressure was measured under controlled conditions, with participants seated and rested for over 20 min.

Biochemical analyses were performed on blood samples collected after a 12 h fast. Lipid profiles, glucose, and creatinine concentrations were measured with automated clinical chemistry analyzers (Hitachi 7600 Automatic Analyzer; Tokyo, Japan). White and RBC blood cell counts were analyzed with Hematology analyzers (DxH 900, Beckman Coulter; Indianapolis, IN, USA). The measurements of Hemoglobin A1c and high-sensitive C-reactive protein levels were conducted using a VARIANT II Turbo (Bio-Rad Laboratories; Hercules, CA, USA) and specialized enzyme-linked immunosorbent assay (ELISA) kits, respectively.

### 2.3. Obesity Definition and Grouping According to Energy Intake

Obesity was defined using the World Health Organization’s Asian-specific criteria: a BMI of ≥25 kg/m^2^ and a waist circumference of ≥90 cm for men and ≥85 cm for women [10]. Participants were divided into four distinct groups based on their obesity status and daily energy intake about their estimated energy requirement (EER) [17]. Energy intake was classified as low or high, with the cutoff set at 100% of the EER. The groups were as follows: (1) low energy intake and lean (L-EN-Lean, n = 24,483); (2) low energy intake and obese (L-EN-Obs, n = 11,729); (3) high energy intake and lean (H-EN-Lean, n = 11,382); and (4) high energy intake and obese (H-EN-Obs, n = 5523).

### 2.4. Dietary Assessment and Nutritional Analysis

Dietary intake was assessed using a semi-quantitative food frequency questionnaire (SQFFQ) developed specifically for the Korean population. The SQFFQ captured details of the intake of 106 food items over the previous six months. Nutrient intake was calculated using CAN-Pro version 3.0 (a computer-aided nutritional analysis program, developed by the Korean Nutrition Society). The intake of nutrients, specifically antioxidants, and bioactive compounds was estimated.

Factor analysis with principal component analysis (PCA) and varimax rotation identified four distinct dietary patterns. The study used eigenvalues > 1.5 and factor-loading values ≥ 0.40 to determine significant dietary pattern contributions [14]. The identified patterns included the Korean balanced diet (KBD), plant-based diet (PBD), Western-style diet (WSD), and rice-based diet (RBD), characterized by specific food group compositions.

### 2.5. Genotyping and Genetic Variant Analysis

Genomic DNA was extracted from the whole blood samples of participants and genotyped using the Korean Chip (Affymetrix, Santa Clara, CA, USA), which focuses on disease-related single nucleotide polymorphisms (SNPs). Stringent quality control measures were applied, ensuring a genotyping accuracy of >98%, missing call rates of <4%, and adherence to the Hardy–Weinberg equilibrium (HWE, *p* > 0.05) with a minor allele frequency (MAF) > 1% [10].

A GWAS was conducted to identify the genetic variants associated with obesity risk, stratified by energy intake levels. The analysis utilized multiple bioinformatics tools, including PLINK version 2.0 for genetic variant analysis, g:Profiler for gene annotation, and the Human Genome Epidemiology (HuGE) Navigator for literature-based gene selection.

### 2.6. Genetic Interaction and Risk Score Modeling

A sophisticated approach was employed to identify and validate genetic interactions using generalized multifactor dimensionality reduction (GMDR) [10]. The analysis selected potential SNP interactions through a rigorous process that included ten-fold cross-validation and consideration of multiple covariates, such as age, gender, lifestyle, and environmental factors.

A polygenic risk score (PRS) was developed to quantify the genetic predisposition to obesity. The scoring methodology involved summing the number of risk alleles across the selected SNPs, with participants categorized into low-, moderate-, and high-risk groups based on their cumulative genetic risk profile [14].

### 2.7. Collection and Screening of Bioactive Compounds from Foods with Low Binding Energy with Brain-Derived Neurotrophic Factor (BDNF) rs6265

A comprehensive investigation of the *BDNF* gene variant rs6265 (also called ‘Val66Met’) was conducted to focus on its missense mutation. Protein structural analysis was performed by obtaining the wild-type protein structure from the UniProt database. Using the Swiss-Protein Data Bank (PDB) Viewer (SPDBV) program, we generated a mutant protein model by precisely introducing the specific amino acid change associated with the genetic variant. A comprehensive molecular database was assembled using the FooDB platform, encompassing approximately 20,000 food-derived compounds as potential ligands [18]. Molecular preparation involved sophisticated computational techniques implemented through MGLTools 1.5.6, a companion software for AutoDock Vina version1.1.2. The preparation process integrated critical molecular modification steps, including hydrogenation of molecular structures, assignment of Kolman charges to nonpolar hydrogen atoms, and conversion of standard PDB file formats to the Protein Data Bank with Partial Charge (Q) and Atom Type (T) (PDBQT) format to ensure optimal compatibility with molecular docking software [18]. Active site identification was performed meticulously using the ProteinsPlus web platform (https://proteins.plus, accessed on 10 March 2024), with a specific focus on the functional pocket containing the mutation site. Molecular docking procedures were executed systematically, screening each food-derived compound against both wild-type and mutated BDNF protein structures. A stringent binding energy threshold of less than −10 kcal/mol was applied to identify the most promising molecular interactions, with lower binding energies indicating more favorable protein–ligand interactions [18].

### 2.8. Clustering of the Natural Compounds with Lower Binding Energy

Natural compounds with low binding energy were subjected to advanced clustering techniques to explore their structural and functional relationships. We employed two complementary clustering approaches: K-means and uniform manifold approximation and projection (UMAP) [19]. The optimal number of clusters was determined using the elbow method and silhouette score analysis. To identify the most representative natural compounds (NCs) within each cluster, we utilized the maximum common substructure (MCS) method, which allowed us to extract the most characteristic molecular features from each identified cluster [19].

In the final analysis, the UMAP-learn package (version 0.2.0) for advanced data visualization and pattern recognition was utilized. The bioactivity data were carefully normalized to ensure equal feature contribution, with the analysis configured to capture local data structures while maintaining global relationships [19]. Specific parameters included 15 nearest neighbors, a minimum distance of 0.1, and projection into a two-dimensional space. Euclidean distance metrics were employed to calculate inter-point distances, enabling a comprehensive visualization that revealed clusters and patterns among natural compounds based on their binding characteristics with wild-type and mutated BDNF proteins.

### 2.9. Statistical Analysis

Statistical analyses were performed using SAS software (version 9.3; SAS Institute, Cary, NC, USA). Two distinct group classifications were utilized to investigate the study objectives comprehensively: (1) four groups stratified by energy intake (low and high, based on EER) and obesity status (lean and obese) and (2) three groups categorized by polygenic risk scores (PRSs: low, medium, and high). For the four-group analysis based on energy intake and obesity status, categorical variables such as gender, education level, and smoking status were assessed using frequency distribution analysis, with statistical significance evaluated via chi-squared tests. Continuous variables (e.g., age, biochemical markers) were presented as means with standard deviations, stratified by the four groups. Group differences were analyzed using a two-way analysis of variance (ANOVA), with covariate adjustments to account for potential confounding factors. Post hoc multiple comparisons were performed using Tukey’s test to identify specific intergroup differences.

The three PRS groups were analyzed using one-way ANOVA to examine differences in continuous variables across low-, medium-, and high-PRS categories. Post hoc pairwise comparisons between PRS groups were also conducted using Tukey’s test.

Logistic regression models were employed to assess the association between PRS and obesity risk, with stratification by energy intake. The first model was adjusted for demographic covariates, including gender, age, residential area, education level, and income. The second model further included lifestyle factors such as smoking status, alcohol consumption, daily energy intake, exercise habits, and medication use for metabolic diseases.

A multivariate interaction model was constructed to evaluate potential interactions between PRS and antioxidant nutrient intake, with participants dichotomized into high- and low-intake groups based on predefined classification criteria. Comprehensive covariate adjustments were applied. Adjusted odds ratios (ORs) and 95% confidence intervals (CIs) were calculated to elucidate the relationships between genetic risk, nutrient intake, and obesity outcomes.

## 3. Results

### 3.1. Baseline Characteristics of the Participants

BMI was significantly higher in the obesity groups (L-EN-Obs and H-EN-Obs; 27.0–27.2 kg/m^2^) than in the lean groups (L-EN-Lean and H-EN-Lean; 22.4 kg/m^2^), regardless of energy intake. Energy intake was significantly higher in the H-EN-Lean and H-EN-Obs groups (128% and 130% of EER, respectively) than in the L-EN-Lean and L-EN-Obs groups (80.3% and 81.6% of the EER, respectively). Participants in the low-energy-intake groups were younger (52.2 and 52.9 years for lean and obese groups) than those in the high-energy-intake groups (56.2 and 57.3 years) (Table 1). The L-EN-Lean group had the highest proportion of men (52.2%), while educational attainment and income levels were consistently higher in the lean groups than in the obese groups, regardless of energy intake. Smoking prevalence was higher in the obese groups than in the lean groups, with 4.61% of participants in the L-EN-Obs group and 2.63% in the H-EN-Obs group. Alcohol consumption was greatest in the H-EN-Obs group, with an average intake of 132 g/week, followed by the H-EN-Lean group (123 g/week), and the lowest in the L-EN-Lean group (101 g/week). Exercise levels were similar between the L-EN-Lean and L-EN-Obs groups (53.4%) but were significantly lower in the H-EN-Obs group than in the H-EN-Lean group (Table 1).

### 3.2. Anthropometric Association

**Figure 1:**
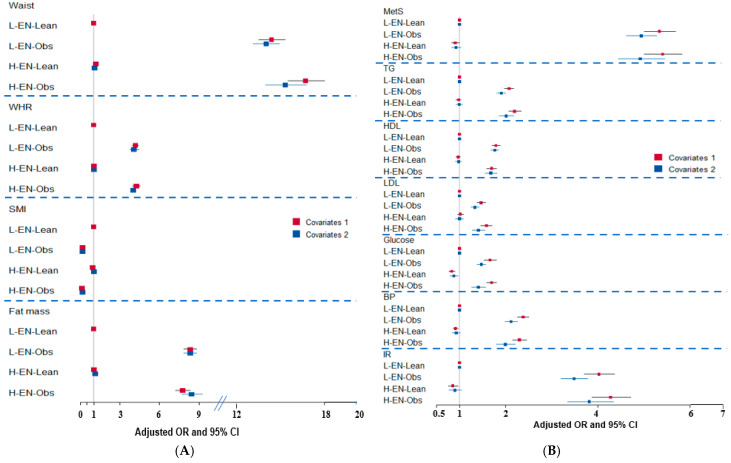
(**A**) Association of body composition in obese and lean individuals based on energy intake. (**B**) Association of metabolic syndrome (MetS) and its components in lean and obese individuals according to energy intake. L-EN-Lean, energy intake lower than estimated energy requirement and lean; L-EN-Obe, low energy intake and obese; H-EN-Lean, high energy intake and lean; H-EN-Obe, high energy intake and obese. Cutoffs: waist circumference > 90 cm for men and >85 cm for women; ratio of waist circumference and hip circumference (WHR) > 0.9 for men and >0.85 for women; skeletal muscle index (SMI) < 0.789 for men and <0.512 for women; fat mass; fat mass 25% for men and 35% for women; serum triglyceride (TG) > 150 mg/dL; serum HDL < 40 mg/dL for men and HDL < 50 mg/dL for women; serum LDL > 160 mg/dL; BP, either of SBP > 140 mmHg or DBP > 90 mmHg; insulin resistance (IR) determined by HOMA-IR > 2.5. OR, odds ratio; CI, confidence intervals; Covariates: Residence area, gender, age, and education for covariate set 1 and the covariates 1 plus energy intake, alcohol intake, physical activity, smoking status, and medication for metabolic diseases for covariate 2.

Body fat percentage was markedly higher in the obesity groups (32.1–32.3%) than in the lean groups (26.6%) (Table 2). Waist and hip circumferences demonstrated notable differences between the lean and obesity groups, while the variations between low- and high-energy-intake subgroups were minimal. After adjusting for covariates (covariate 2), waist circumference, fat mass, and fat mass index were significantly associated with obesity in both low and high-energy-intake subgroups. Specifically, waist circumference was 17.5- and 18.6-fold higher in the L-EN-Obs and H-EN-Obs groups than the L-EN-Lean group, respectively, while fat mass was 4.558- and 5.24-fold higher in the same groups (Figure 1A). In contrast, SMI exhibited an inverse relationship with fat mass, showing significantly lower values in the L-EN-Obs and H-EN-Obs groups than the L-EN-Lean group, with reductions of 0.137- and 0.152-fold, respectively, after adjusting for covariate 2. No significant associations were observed for waist circumference, fat mass, or SMI in the H-EN-Lean group relative to the L-EN-Lean group (Figure 1A). These results underscored the critical role of obesity status in driving anthropometric differences over energy intake variations.

### 3.3. Risk of Metabolic Syndrome

The L-EN-Obs (OR = 4.94) and H-EN-Obs (OR = 4.92) groups exhibited significantly higher adjusted ORs for MetS than the L-EN-Lean group. In contrast, the OR for the H-EN-Lean group did not significantly differ from that of the L-EN-Lean group (Figure 1B). These findings suggest that obesity, irrespective of energy intake levels, was a stronger determinant of MetS risk than energy intake alone. Further analysis revealed that the L-EN-Obs and H-EN-Obs groups were significantly associated with all MetS components compared to the L-EN-Lean group. These included waist circumference (17.0- and 18.6-fold higher, respectively), serum glucose (1.47- and 1.41-fold higher, respectively), hypo-HDL cholesterol (1.76- and 1.68-fold higher, respectively), LDL cholesterol (1.33- and 1.41- fold higher, respectively), triglyceride concentrations (1.91- and 2.01-fold higher, respectively), insulin resistance (3.49- and 3.81-fold higher, respectively), and blood pressure (2.11- and 2.0-fold higher, respectively) (Figure 1B). Notably, there were no significant associations between the H-EN-Lean and L-EN-Lean groups for MetS or its components.

### 3.4. Intake of Antioxidant Nutrients and Bioactive Compounds

Energy intake was significantly higher in the H-EN-Lean and H-EN-Obs groups (80.3% and 81.6% of the EER, respectively) than in the L-EN-Lean and L-EN-Obs groups (128% and 130% of EER, respectively) (Table 3). Obese participants had slightly higher energy intake than their lean counterparts within both the low- and high-energy-intake groups. Interestingly, obesity incidence was lower among individuals with a higher intake of plant-based diets (PBDs), regardless of energy intake levels (Table 3). The intake of antioxidant nutrients, including vitamins C, E, and D, and selenium, was significantly higher in the obese groups than the lean groups, particularly in the comparison between the H-EN-Lean and H-EN-Obs groups. Regarding bioactive compounds, the intake of total anthocyanins (a sum of cyanidin, delphinidin, malvidin, pelargonidin, peonidin, and petunidin), especially cyanidin, was higher in the lean groups than in the obese groups, with the H-EN-Lean group consuming more than the L-EN-Obs group (Table 3). Conversely, the intake of flavonoids (a sum of quercetin, luteolin, kaempferol, and apigenin) was significantly higher in the L-EN-Obs group than in the L-EN-Lean group. Isoflavonoid intake varied according to energy intake levels but showed no significant differences between obese and lean groups (Table 3).

### 3.5. Genetic Impact

The statistically significant association of genetic variants with obesity in participants with low energy intake is shown in a Manhattan plot. Significant genetic variants existed in chromosomes 1, 11, 16, and 18 (Figure S1A). A Q-Q plot (Figure S1B) shows the quantile distribution of the log of observed *p* values versus the log of expected *p* values. The lambda value was 1.134. However, there were no genetic variants significantly associated with high energy intake participants (*p* < 5 × 10^−8^). Ten genetic variants with the interactions are presented in Table 4. The genetic variants significantly associated with obesity and low energy intake included SEC16 homolog B, endoplasmic reticulum export factor (*SEC16B* rs506589), brain-derived neurotrophic factor (*BDNF* rs6265), FTO (rs1421085), gastric inhibitory polypeptide receptor (*GIPR* rs1444988703), and *BDNF* Antisense RNA (*BDNF-AS* rs925947) (*p* < 5 × 10^−8^). To make the PRS model satisfy the criteria, adenylate cyclase 3 (ADCY3 rs1965122), polybromo 1(*PBRM1* rs73078824), glutaminyl-peptide cyclotransferase like (*QPCTL* rs9636135), symplekin scaffold protein (*SYMPK* rs10408067), and divergent-paired related homeobox (*DPRX* rs796090051) were added in the PRS model at liberal significance (P5 × 10^−7^) (Table 4). They met the conditions of MAF > 1% and *p*-value for HWE > 0.05.

**Figure 2:**
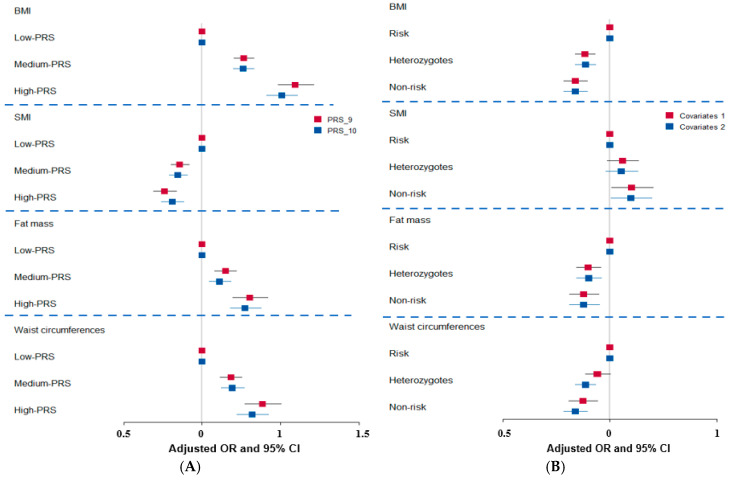
(**A**) Association of body composition with genetic variants and their polygenic risk score (PRS) with 9 genetic variants. (**B**) Association of body composition with brain-derived neurotrophic factor (*BDNF*) rs6265 genetic variants. OR, odds ratio; CI, confidence intervals; covariates: residence area, gender, age, and education for covariate set 1 and the covariates 1 plus energy intake, alcohol intake, physical activity, smoke, and medication for metabolic diseases for covariate 2. The cutoffs of BMI, SMI, fat mass, and waist circumferences were used shown in the Figure 1 legend.

The best models with interactions between the genetic variants influencing obesity with low energy intake were selected when satisfying *p*-value < 0.05 for the sign test of testing balanced accuracy (TEBA) and cross-validation consistency (CVC) 10/10. The models to meet the criteria included 9- and 10-SNPs (Supplementary Table S1). The PRS with 9 and 10 genetic variants showed similar patterns, but the PRS with 9 genetic variants showed greater ORs for BMI (1.59), fat mass (1.30), and waist circumferences (1.38) compared to those with 10 genetic variants. The SMI had an inverse association with the PRS with 9 and 10 genetic variants (Figure 2A). The PRS with nine SNPs showed that BMI, waist and hip circumferences, and WHR were significantly higher in the high-PRS group than in the low-PRS groups. In contrast, the SMI was lower in the high-PRS group than in the low-PRS group. Among MetS and its components, MetS incidence, systolic blood pressure (SBP), and diastolic blood pressure (DBP) were higher in the high-PRS group than in the low-PRS group.

Since *BDNF* rs6265 was a missense mutation, it was used for further analysis to find bioactive compounds. BMI, waist circumference, and body fat mass were higher in the risk allele group than in the non-risk allele group. On the other hand, SMI was lower in the risk allele group than in the non-risk allele group (Table 5). The *BDNF* rs6265 non-risk allele was positively associated with BMI, waist circumference, and body fat mass; heterozygotes and minor alleles were positively associated and SMI was inversely associated with obesity (Figure 2B).

### 3.6. Interaction of BNDF Val66Met Polymorphisms with Food Intake Influencing Obesity

**Figure 3:**
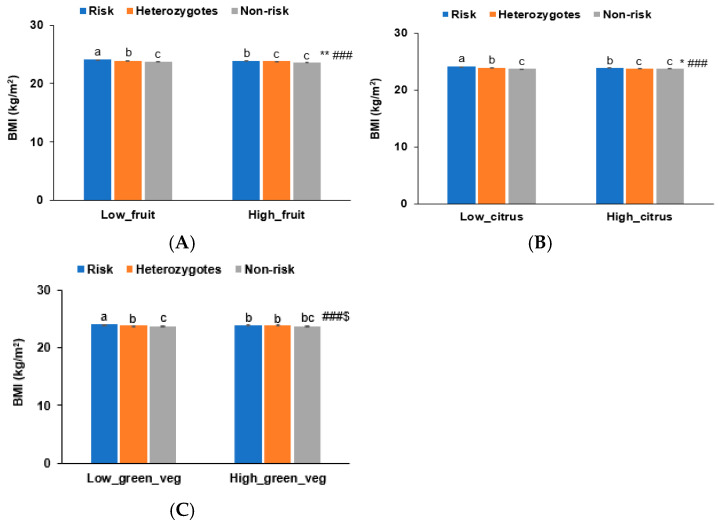
Body mass index interacting brain-derived neurotrophic factor (*BDNF*) rs6265 with antioxidants in participants with low energy intake. (**A**) Fruit intake (cutoff: 2.5 servings/day, including fruit juice). (**B**) Citrus fruits (cutoff: 1 serving/day). (**C**) Green vegetables (green_veg; cutoff: 1 serving/day). * Significantly different by fruit intake at *p* < 0.05. ** Significant difference by citrus fruit intake at *p* < 0.01. ### Significantly different by *BDNF* rs6265 alleles. $ Significant interaction between green vegetable (veg) intake and BDNF rs6265 at *p* < 0.05. a–c Different letters on the bar indicated significant differences among the groups by Tukey test at *p* < 0.05.

Consistent with the binding energy analysis of BNDF Val66Met WT and MT with bioactive compounds, BMI was lower in the high-fruit-intake group compared to the low-fruit-intake group (<2.5 serving/day, including fruit juice) (*p* < 0.01; Figure 3A), regardless of BDNF Val66Met, after adjusting for age, gender, education, income, alcohol intake, smoking status, and exercise. Citrus fruit intake with a cutoff of 1 serving/day showed a trend similar to fruit intake (Figure 3B). BMI did not differ with green vegetable intake in both BDNF Val66Met WT and MT. However, BMI and green vegetable intake interacted with BDNF Val66Met WT and MT (Figure 3C). In high-green-vegetable-intake (≥1 serving/day) subjects, BMI was similar among the BNDF alleles, while BMI was lower in subjects with the non-risk allele than in those with the risk allele in the low-vegetable-intake group (Figure 3C).

### 3.7. Bioactive Compounds with Low Binding Energy with Wild-Type (WT) and Mutant-Type (MT) BDNF Val66Met (rs6265)

**Figure 4:**
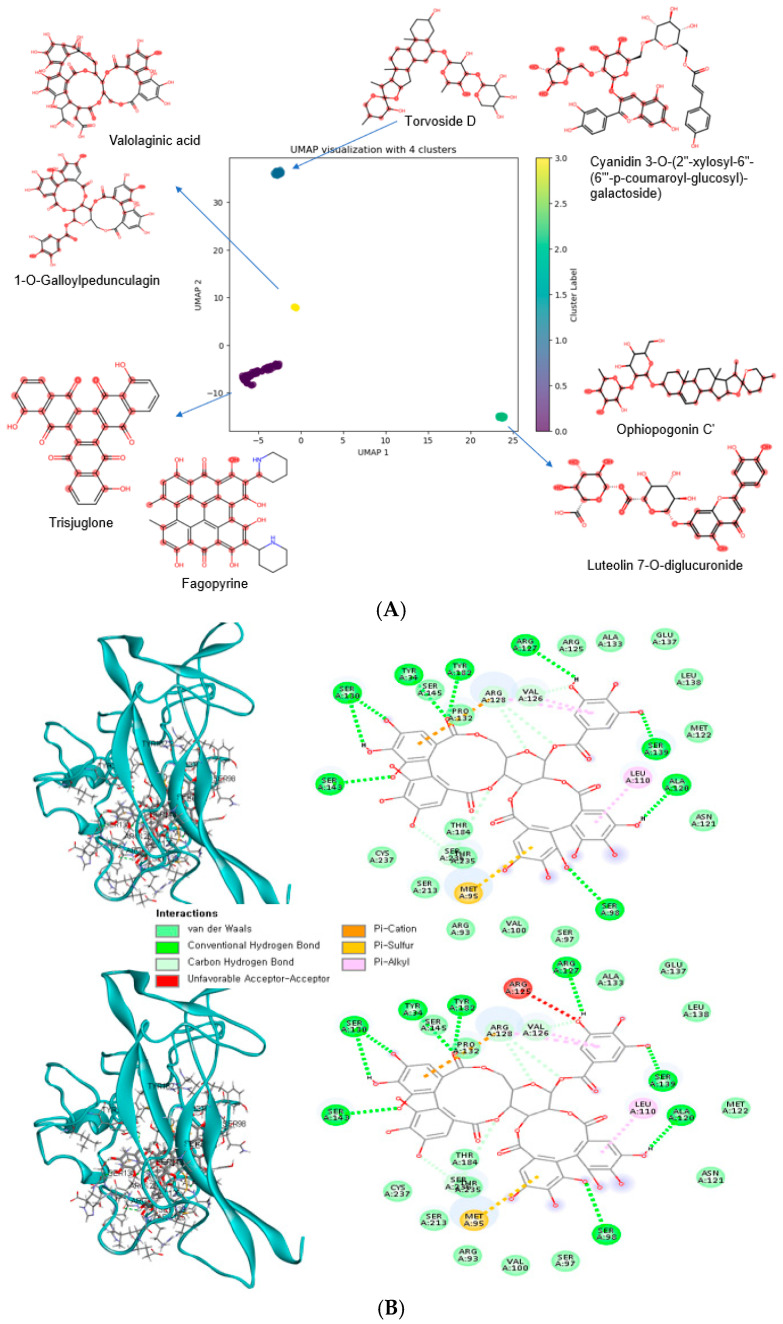

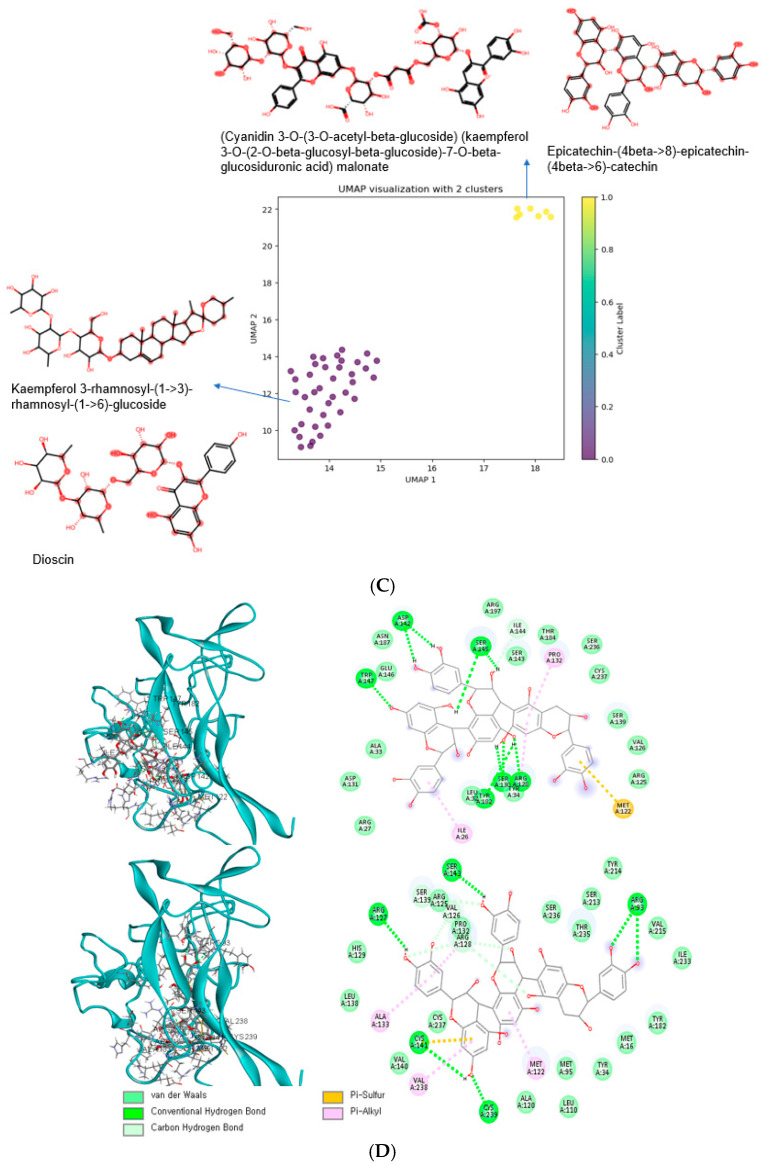

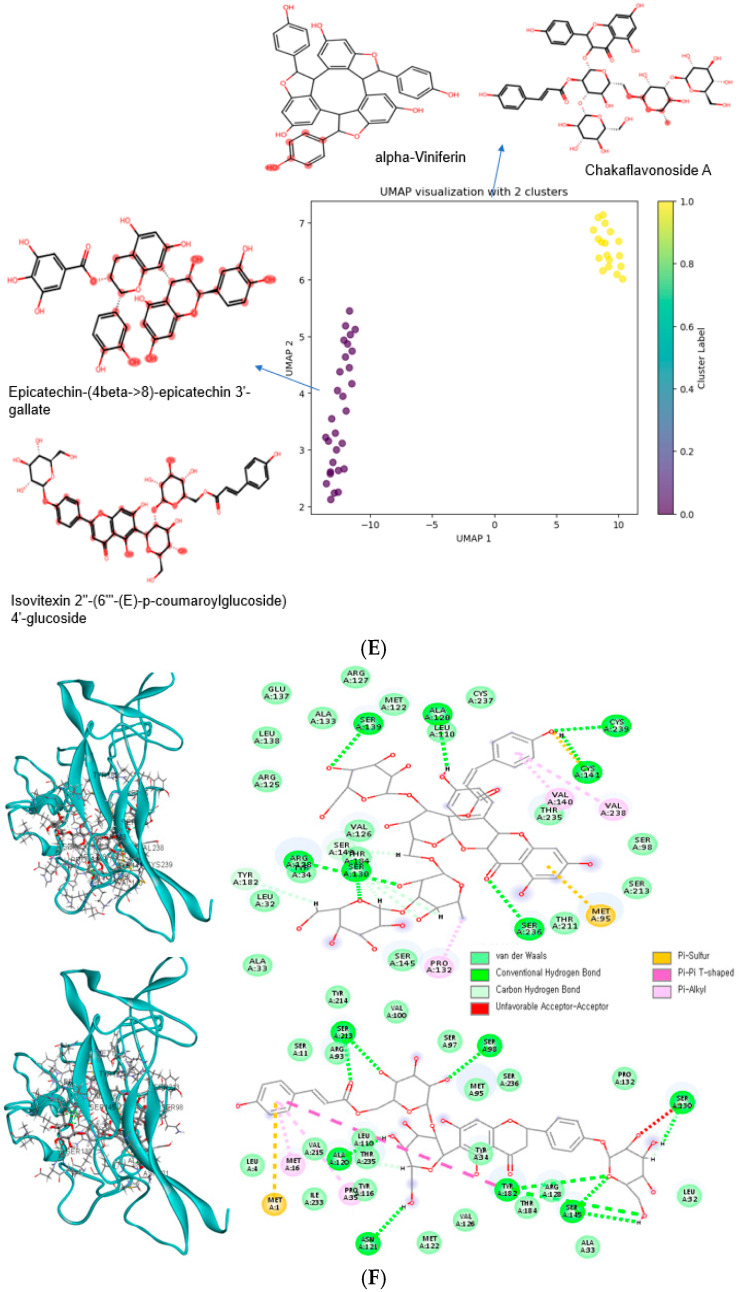
Uniform manifold approximation and projection (UMAP) analysis of natural compounds with a binding energy of lower than −10 kcal/mol to brain-derived neurotrophic factor (BDNF) Val66 Met (rs 6265) and maximum common substructures of each cluster. (**A**) Natural compounds (NCs) lower than −10 kcal/mol in both wild-type (WT) and mutated type (MT) of BDNF Val66Met. (**B**) Diagrammatic representation and 2D depiction of 1-O-galloylpedunculagin (ball and stick model) binding with WT (upper) and MT proteins (lower) of BDNF Val66Met. (**C**) NCs lower than −10 kcal/mol in the WT of BDNF Val66. (**D**) Diagrammatic representation and 2D depiction of epicatechin-(4beta->8)-epicatechin-(4beta->6)-catechin binding with WT (upper) and MT proteins (lower) of BDNF Val66Met. (**E**) NCs lower than −10 kcal/mol in the MT of BDNF 66Met. (**F**) Diagrammatic representation and 2D depiction of chakaflavonoside A binding with WT (upper) and MT (lower) proteins of BDNF Val66Met.

We evaluated the binding energies of the WT and MT polymorphisms of BDNF Val66Met with 197 bioactive compounds to determine their potential for modulating BDNF activity. Among the tested compounds, 107 showed strong binding (<−10 kcal/mol) to both wild-type and mutant variants, while 45 compounds bound selectively to WT and another 45 to MT. Each selected compound was characterized by unique structural features, offering insights into the molecular basis of their interaction with BDNF. The bioactive compounds with binding energy <−10 kcal/mol were categorized into three groups: those binding to both wild-type (WT) and mutant-type (MT), WT only, or MT only. Table 6 and Figure 4 present the UMAP clustering with MCS analysis results for each group. In the NC lowering binding energy with both WT and MT (Figure 4A,B), cluster 1 (binding energy <−11 kcal/mol) comprised bioactive compounds including valolaginic acid, 1-O-galloylpedunculagin, potentillin, casuarinin, and pipercyclobutanamide B (Figure 4A). These compounds were predominantly found in guava, pomegranate, herbs, spices, and fruits. The specific chemical structures of these NCs, as revealed by the MCS analysis, suggest unique molecular interactions with the BDNF protein (Table 6 and Figure 4A). The profile was expanded in cluster 2 to include trisjuglone, casuarinin, stachyurin, asterlingulatoside D, fagopyrin, and others, with additional sources such as common buckwheat, common walnut, and nuts (Table 6 and Figure 4A). Cluster 3 mainly included compounds with a binding energy of −10.1 to −10.2 kcal/mol, such as kaempferol 3-O-rhamnosyl-rhamnosyl-glucoside, punicafolin, torvoside D, cyanidin 3-O-(2″-xylosyl-6″-(6‴-p-coumaroyl-glucosyl)-galactoside), and others. Cluster 4 included compounds with a binding energy of −10 kcal/mol, such as luteolin 7-O-diglucuronide, ophiopogonin C′, theaflavin 3,3′-gallate, anigorootin, and others (Table 6 and Figure 4A). 1-O-galloylpedunculagin had 8 hydrogens around the BDNF WT and MT (Figure 4B).

Our analysis of natural compounds for WT only identified several specific bioactive compounds with low binding energy with BDNF WT protein (Val66) (Figure 4C). They had similar binding energy with BDNF WT and MT protein of between −10 and −10.9 kcal/mol and they were clustered into two clusters with UMAP (Table 6; Figure 4C). These compounds were cyanidin and epicatechin, kaempferol and its glycosides, pitheduloside K, and others in cluster 1, and kaempferol 3-rhamnosyl-(1->3)-rhamnosyl-(1->6)-glucoside, dioscin, hesperidin, beta1-chaconine, and others in cluster 2 (Table 6; Figure 4C). These compounds originated from diverse sources, such as the common grape, citrus fruits, onion, yam, pithecellobium dulce seeds, and chives. In Figure 4D, the binding energy between epicatechin-(4beta->8)-epicatechin-(4beta->6)-catechin and the BDNF WT protein (six hydrogen bonds near the BDNF WT) was lower, with more hydrogen bonds than the MT protein (four hydrogen bonds but less tight to the BDNF MT).

In contrast, compounds with lowering binding energy between −13.9 and −10 kcal/mol exclusively with the BDNF MT polymorphism (66Met) were also identified (Figure 4E). These were alpha-viniferin, quillaic acid and its glycosides, tragopogonsaponin Q, ceposide D, eleutheroside M, cinnamtannin A2, and chakaflavonoside A in cluster 1. Epicatechin glycated compounds and acutoside G were in cluster 2 in the UMAP clusters (Table 6; Figure 4E). These compounds were detected in common grapes, green vegetables, onion, cocoa beans, cinnamon, tea, and buckwheat. In Figure 4F, the binding energy between chakaflavonoside A and the BDNF MT protein (eight hydrogen bonds near the BDNF MT) was lower, with more hydrogen bonds than the WT (six hydrogen bonds but less tight to the BDNF WT). MCS analysis of the UMAP clusters not only identified these compounds but also provided visual representations of their specific chemical structures. This structural visualization is crucial for understanding the potential molecular mechanisms of the BDNF interaction with the natural compounds, highlighting the unique chemical features that may contribute to their binding properties. This approach provides a comprehensive understanding of the structural determinants driving compound–protein interactions, surpassing conventional binding energy analysis by elucidating the molecular mechanisms underlying these interactions.

**Figure 5:**
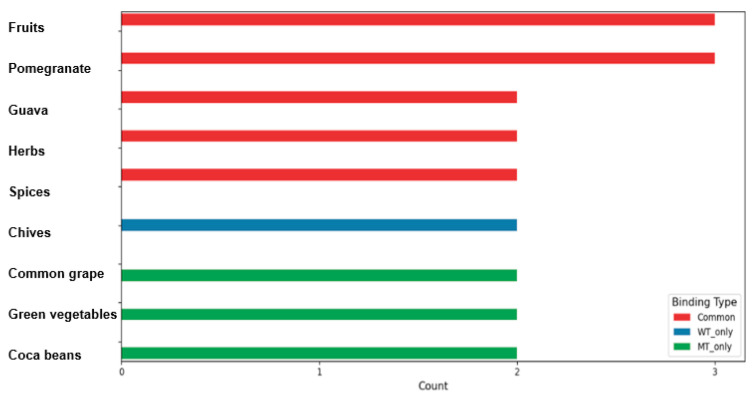
Distribution and overlap of natural compounds in various food sources.

Food sources with the most diverse bioactive compounds affecting the BDNF Val66Met binding energy were carefully mapped. Fruits, especially guava and pomegranate, along with herbs and spices, demonstrated low binding energy with both WT and MT protein variants (Figure 5). Interestingly, chives showed low binding energy with WT protein but not MT, while tea, common grapes, green vegetables, and cocoa beans exhibited low binding energy with MT protein but not with WT. These comprehensive findings suggest that certain fruits, particularly guava and pomegranate, could potentially modulate BDNF protein binding, potentially influencing BDNF activity and the related physiological processes. The intricate interactions revealed through the MCS analysis highlighted the complex role of dietary components in molecular interactions and potential neurobiological mechanisms shown in the previous studies [20,21].

## 4. Discussion

Although numerous genetic variants linked to obesity risk have been identified [10,22,23], translating this knowledge into clinical practice and public health prevention strategies remains challenging. Managing energy balance is fundamental to body weight regulation. However, individual variability in response to energy intake highlights the role of genetic predisposition. Previous studies have shown that genetic variants in antioxidant-related pathways significantly influence metabolic outcomes, with polymorphisms in oxidative stress genes (such as superoxide dismutase 2 and glutathione peroxidase) modifying the relationship between dietary components and MetS risk factors [24,25,26]. These genetic variations can affect how individuals respond to different dietary patterns and dietary bioactive components, particularly in BMI and MetS components.

The present study offers novel insights into how the interaction of genetic predisposition, energy intake, and nutrient intake influences obesity risk. Notably, our demographic analysis revealed that lean groups showed higher educational attainment and income levels regardless of energy intake, consistent with previous research on social determinants of obesity. Higher socioeconomic status is often associated with better nutritional knowledge, access to healthier food options, and greater resources for physical activity [27]. The higher proportion of males in the L-EN-Lean group may reflect sex-specific differences in energy metabolism, body composition, dietary patterns, physical activity, and alcohol drinks [28,29]. Beyond these sociodemographic factors, we observed that genetic variants such as BDNF rs6265 had a greater impact on obesity in individuals with a lower energy intake, while their effects diminished in those with a higher energy intake. Molecular docking revealed the reduced binding energy of BDNF Val66Met with bioactive compounds in citrus fruits, green vegetables, cocoa beans, and herbs, suggesting a potential mechanism for the modulation of genetic factors through the diets. Cohort analyses further validated these interactions. The intake of citrus fruits and green vegetables was inversely associated with obesity risk in participants carrying the BDNF rs6265 variant. In the high-energy-intake group, antioxidant-rich diets—particularly those containing compounds such as cyanidin and epicatechin—were inversely associated with obesity, independent of genetic predisposition. These findings underscore the intricate relationships between genetics, diet, and lifestyle and highlight the potential for personalized dietary interventions targeting genetic susceptibilities to mitigate obesity risk.

Obesity is a complex polygenic trait, influenced by numerous genetic variants, each having a small effect that interacts with lifestyle factors. One of the highlights of our study is that genetic predisposition to obesity was primarily evident in individuals with low energy intake, as no significant genetic associations were observed in the high-energy-intake group at a threshold of *p* < 5 × 10⁻^8^. This suggests that genetic variants, such as *BDNF rs6265*, *FTO rs1421085*, and *SEC16B rs506589*, are more likely to predispose individuals to obesity when their energy intake is below the EER. These findings align with previous studies demonstrating the role of *FTO* in energy expenditure, with its reduced activity leading to decreased adiposity independent of energy intake [30], and *BDNF* in mediating fat utilization via the sympathetic nervous system, impacting energy expenditure [31].

Notably, in the low-energy-intake group, the genetic variants associated with obesity overlapped with those identified in broader population-based studies [10,11]. However, their effects were diminished in individuals with high energy intake, indicating that lifestyle factors, such as energy intake and physical activity, may mitigate genetic risk. *FTO* rs1421085 has been shown to interact with physical activity to modulate obesity risk in Caucasian populations [32], and *BDNF* rs6265 influences dietary preferences and satiety regulation [33]. Additionally, *SEC16B* variants are associated with impaired lipid absorption, which may protect against high-fat-diet-induced obesity [23,34], consistent with our observations in the low-energy-intake group.

While PRSs that incorporate significant and moderately significant variants did not show interactions with nutrient intake or lifestyle factors in the low-energy-intake group, they demonstrated interactions with lifestyle factors such as physical activity, alcohol intake, and plant-based diets in the broader cohort. This supports findings from previous studies that emphasize the importance of gene–environment interactions in obesity risk modulation [11,35]. Collectively, our findings highlight the complex interplay of genetic predispositions, energy intake, and lifestyle factors in determining obesity risk, providing valuable insights for personalized prevention strategies.

Genetic insights from gene discovery efforts are increasingly applied to precision medicine, particularly for predicting obesity risk and identifying potential therapeutic targets [23]. While monogenic obesity treatments, such as injectable melanocortin-4 receptor (*MC4R*) agonists, have shown efficacy in addressing mutations like those in leptin receptors (*LEPR*), such interventions are less applicable to polygenic obesity, which involves numerous variants with small cumulative effects [23]. For polygenic variants, oral treatments or dietary interventions tailored to genetic predispositions may offer a more feasible and effective approach [36]. Our findings highlight the potential of *BDNF* rs6265, a missense mutation, as a critical target for dietary interventions. We identified 107 bioactive compounds with strong binding affinity (binding energy < −10 kcal/mol) to both wild-type and mutant variants of BDNF Val66Met, many of which are present in commonly consumed foods such as citrus fruits (e.g., pomegranate, orange, lemon), guava, herbs, and spices. Previous studies have demonstrated that pomegranate extract significantly increases BDNF expression in hippocampal neurons [37], while guava’s polyphenolic compounds have been shown to enhance BDNF signaling pathways in preclinical models [38,39]. The neuroprotective effects of these fruits have been attributed to their rich content of specific flavonoids including anthocyanins that can cross the blood–brain barrier and directly modulate BDNF expression [40]. Interestingly, compounds found in green vegetables, grapes, and cocoa beans exhibited a stronger binding affinity specifically to the mutant variant. These results align with prior research [41] suggesting that BDNF plays a central role in energy expenditure, neuronal plasticity, and fat utilization through the sympathetic nervous system.

Importantly, our findings highlight the context-dependent nature of genetic impact. While genetic variants like *BDNF* rs6265 significantly influenced the obesity risk in individuals with a lower energy intake, their effects were negligible in the high-energy-intake groups. This observation emphasizes the importance of stratifying populations by dietary energy intake when exploring gene–environment interactions, a key factor often overlooked in prior studies [36]. Moreover, our study supports the growing evidence for diet-gene interactions, building upon previous work that highlights the role of polygenic variants in modulating obesity risk through mechanisms such as energy expenditure, lipid absorption, and energy intake [22,23,35]. By demonstrating that natural compounds can influence the function of *BDNF* rs6265, our findings pave the way for genotype-informed dietary interventions. This approach could complement existing treatments, offering a personalized and practical strategy to mitigate obesity risk, particularly in populations with specific genetic predispositions.

Validation of in silico findings with cohort data is critical for translating molecular insights into actionable health strategies. While prior studies have highlighted the metabolic benefits of citrus fruits, green vegetables, and other antioxidant-rich foods due to their anti-inflammatory and antioxidant properties [8,9,42], few have directly linked these dietary components to specific genetic factors associated with obesity [43]. Our study bridges this gap by demonstrating that natural compounds such as valolaginic acid, torvoside C, citrusin II, and rugosin E (with binding energies below −11 kcal/mol) interact with both WT and MT BDNF proteins in silico. These compounds were identified in foods such as citrus fruits, guava, herbs, and spices for WT and MT and in common grapes, green vegetables, and cocoa beans for MT alone. Notably, our findings differ from prior studies, such as those reporting weaker binding affinities (−4.5 to −6.7 kcal/mol) for compounds like vitamin D3, curcumin, vitamin C, and quercetin with BDNF protein [44]. We excluded these compounds due to their higher binding energy, focusing instead on those meeting our stricter criteria. Through this approach, we identified citrus fruits and green vegetables as the most promising dietary sources for influencing *BDNF* rs6265, findings validated through city-based cohort data. In the cohort, green vegetable and citrus fruit intake was inversely associated with obesity risk, particularly in individuals carrying the *BDNF* rs6265 variant, consistent with the molecular docking results. These results extend the prior research on *BDNF* rs6265, which has shown its role in modulating energy expenditure and fat metabolism, by providing robust evidence for dietary modulation of its effects [45,46]. This integration of molecular and cohort data underscores the potential for personalized dietary interventions targeting the genetic risk factors for obesity.

Further, our study demonstrated that *BDNF* rs6265 exhibits variant-specific binding affinity to antioxidant compounds. Green vegetable-derived compounds showed stronger interactions with the mutant allele, aligning with cohort data, where green vegetable intake was inversely associated with obesity risk in carriers of the mutant allele, specifically within the low-energy-intake group. This finding suggests a gene–diet interaction that modulates obesity risk under restricted energy intake. In contrast, no genetic impact was observed in the high-energy-intake group. However, in this group, higher antioxidant intake—specifically vitamin C, vitamin D, vitamin E, total anthocyanins, and isoflavonoids—was associated with leanness. These findings align with prior studies [47,48] which showed that antioxidants mitigate oxidative stress and inflammation, which are the key drivers of obesity by increasing adipocyte number and size, promoting lipogenesis, and stimulating preadipocyte differentiation under conditions of elevated oxidative stress, such as high energy intake. Our study adds to this body of literature by demonstrating that genetic susceptibility to obesity may be masked in high-energy environments, where the protective effects of antioxidant-rich diets dominate. These findings highlight the importance of tailoring dietary recommendations based on both genetic predispositions and energy intake levels. By highlighting gene–diet interactions, particularly under varying energy intakes, this study provides a foundation for developing personalized dietary interventions to optimize obesity prevention and management strategies.

This study provides novel insights into the interplay between genetic predisposition, energy intake, and antioxidant-rich diets in modulating obesity risk. Specifically, it highlights that genetic variants such as *BDNF* rs6265 and *FTO* rs1421085 are primarily associated with obesity risk in individuals with low energy intake. Antioxidant nutrients, such as vitamins C, D, and E, and bioactive compounds like anthocyanins and isoflavonoids, are more relevant in the context of high energy intake. Using molecular docking to identify variant-specific binding affinities of bioactive compounds, validated with cohort data, represents a novel approach linking in silico findings to real-world dietary and genetic interactions. However, the study has limitations, including the observational nature of the cohort analysis, which limits causal inference, and the lack of experimental validation of the identified effects of bioactive compounds on genetic pathways. Additionally, the reliance on self-reported dietary data may introduce measurement bias, even though the KNIH research team carefully designed, collected, and managed the data with the help of skilled technicians. The findings may not be generalizable to populations with different genetic and dietary backgrounds. Future studies should include functional assays and randomized trials to confirm these interactions and explore the mechanisms further.

## 5. Conclusions

Our study highlights the importance of gene–diet interactions in obesity, particularly those involving the *BDNF* rs6265 variant. Molecular docking and cohort data revealed that polyphenols, particularly flavonoids, in citrus fruits, green vegetables, and cocoa beans demonstrated high binding affinities to BDNF Val66Met, with stronger associations in individuals carrying the mutant allele. These findings suggest that dietary interventions, especially antioxidant intake, tailored to genetic profiles could mitigate obesity risk, especially under restricted energy intake conditions. In contrast, when the energy intake is high, antioxidant-rich diets appeared to play a more dominant role in reducing obesity risk, independent of genetic predisposition. Future research should validate these findings across diverse populations and investigate the long-term effects of personalized dietary interventions in clinical trials. By bridging molecular insights with practical dietary recommendations, our study provides a foundation for advancing precision medicine in obesity prevention and management.

## Figures and Tables

**Table 1:** General characteristics and body composition of the participants according to energy intake and obesity status.

	L-EN-Lean (n = 24,483)	L-EN-Obs (n = 11,729)	H-EN-Lean (n = 11,382)	H-EN-Obs (n = 5523)
BMI (kg/m^2^)	22.4 ± 0.01 ^c^	27 ± 0.02 ^b^	22.4 ± 0.02 ^c^	27.2 ± 0.03 ^a^**^+++##^
Energy intake (EER %)	80.3 ± 0.13 ^d^	81.6 ± 0.19 ^c^	128.4 ± 0.19 ^b^	130.2 ± 0.28 ^a^***^+++#^
Age (years)	52.2 ± 0.05 ^d^	52.9 ± 0.07 ^c^	56.2 ± 0.08 ^b^	57.3 ± 0.11 ^a^***
Gender (male %)	9599 (39.2)	6117 (52.2)	1930 (17.0)	1510 (27.3) ***
Education (N, %)				
<High school	3171 (18.1)	2065 (24.0)	1294 (15.7)	1036 (24.2) ^$$$^
High school	12,978 (74.1)	5955 (69.3)	6362 (77.0)	2942 (69.3)
College and over	1361 (7.77)	576 (6.7)	611 (7.39)	270 (6.36)
Monthly income (N, %)				
≤USD 2000	2416 (10.4)	1312 (11.9)	847 (7.89)	574 (11.2) ^$$$^
USD 2000–4000	9882 (42.5)	4749 (43.0)	4864 (45.3)	2333 (44.7)
>USD 4000	10,967(47.1)	4975 (45.1)	5018 (46.8)	2309 (44.3)
Non-Smoking (N, %)	22,290 (91.1)	10,338 (88.2)	10,940 (96.2)	5169 (93.6) ^$$$^
Former smoking	1224 (5.0)	847 (7.22)	248 (2.18)	206 (3.73)
Current smoking	964 (3.94)	541 (4.61)	188 (1.65)	145 (2.63)
Alcohol (g/week)	101 ± 2.16 ^c^	123 ± 3 ^b^	104 ± 3.48 ^c^	132 ± 4.6 ^a+++^
Exercise (Yes: N, %)	13,042 (53.4)	6254 (53.4)	6552 (57.7)	3013 (54.7) ^$$$^

Values represent adjusted means ± standard errors or number (N) and percentage. L-EN-Lean, energy intake lower than estimated energy requirement and lean; L-EN-Obe, low energy intake and obese; H-EN-Lean, high energy intake and lean; H-EN-Obe, high energy intake and obese. EER, estimated energy requirement. The covariates included age, gender, energy intake, residence area, education, metabolic syndrome, smoking, alcohol intake, fat intake, physical activity, and any medication for metabolic diseases. An independent variable was eliminated from the covariates. ^+++^ Significant difference with obesity by two-way ANOVA at *p* < 0.001. ** Significant difference with energy intake by two-way ANOVA at *p* < 0.01 and *** at *p* < 0.001. ^#^ Significant interaction between energy intake and obesity by two-way ANOVA at *p* < 0.05 and ^##^ at *p* < 0.01. ^a–d^ Different superscript letters indicated significant differences among the groups by Tukey test at *p* < 0.05. ^$$$^ Significantly different among four groups by chi-square test at *p* < 0.05.

**Table 2:** Body composition of the participants according to energy intake and obesity status.

	L-EN-Lean (n = 24,483)	L-EN-Obs (n = 11,729)	H-EN-Lean (n = 11,382)	H-EN-Obs (n = 5523)
Skeletal muscle mass index (kg/m^2^)	7.16 ± 0.004 ^b^	6.67 ± 0.005 ^a^	7.16 ± 0.006 ^b^	6.63 ± 0.008 ^a+++#^
Fat mass index (kg/m^2^)	10.4 ± 0.01 ^c^	12.5 ± 0.01 ^b^	10.4 ± 0.01 ^c^	12.7 ± 0.02 ^a^*^+++#^
Body fat (%)	26.6 ± 0.02 ^c^	32.1 ± 0.02 ^b^	26.6 ± 0.03 ^c^	32.3 ± 0.04 ^a^**^+++##^
Waist circumferences (cm)	77.5 ± 0.04 ^c^	87.4 ± 0.06 ^b^	77.6 ± 0.07 ^c^	87.8 ± 0.09 ^a^**^+++#^
Hip circumferences (cm)	91.9 ± 0.03 ^c^	98.5 ± 0.05 ^b^	92 ± 0.05 ^c^	98.8 ± 0.07 ^a^**^+++##^
WHR	0.84 ± 0.0004	0.89 ± 0.0005	0.844 ± 0.0001	0.89 ± 0.0008 ^+++^

Values represent adjusted means ± standard errors. L-EN-Lean, energy intake lower than estimated energy requirement and lean; L-EN-Obe, low energy intake and obese; H-EN-Lean, high energy intake and lean; H-EN-Obe, high energy intake and obese. WHR, the ratio of waist circumference and hip circumference. The covariates included age, gender, energy intake, residence area, body mass index, education, metabolic syndrome, smoking, alcohol intake, fat intake, physical activity, and any medication for metabolic diseases. An independent variable was eliminated from the covariates. ^+++^ Significant difference with obesity by two-way ANOVA at *p* < 0.001. * Significant difference with energy intake by two-way ANOVA at *p* < 0.05 and ** at *p* < 0.01. ^#^ Significant interaction between energy intake and obesity by two-way ANOVA at *p* < 0.05 and ^##^ at *p* < 0.01. ^a–c^ Different superscript letters indicated significant differences among the groups by Tukey test at *p* < 0.05.

**Table 3:** Participants’ nutrient intake according to energy intake and obesity status.

	L-EN-Lean (n = 24,483)	L-EN-Obs (n = 11,729)	H-EN-Lean (n = 11,382)	H-EN-Obs (n = 5523)
Energy intake (EER %)	80.3 ± 0.13 ^d^	81.6 ± 0.19 ^c^	128.4 ± 0.19 ^b^	130.2 ± 0.28 ^a^***^+++#^
Plant-based diet (Yes, N, %)	5892 (24.1)	2303 (19.6)	6817 (59.9)	2857 (51.7) ^$$$^
Dietary fiber (g/day)	14.6 ± 0.06 ^b^	14.8 ± 0.08 ^ab^	14.7 ± 0.09 ^b^	15.1 ± 0.12 ^a^*^++^
Vitamin C (mg/day)	103 ± 0.39 ^c^	104 ± 0.54 ^c^	112 ± 0.62 ^a^	108 ± 0.82 ^b^***^++###^
Vitamin D (μg/day)	6.33 ± 0.034 ^c^	6.25 ± 0.048 ^c^	6.73 ± 0.055 ^a^	6.53 ± 0.073 ^b^***^++^
Vitamin E (mg/day)	8.02 ± 0.054 ^a^	7.89 ± 0.075 ^ab^	7.84 ± 0.087 ^ab^	7.58 ± 0.115 ^b^*^#^
Selenium (μg/day)	15.1 ± 0.15 ^a^	15.1 ± 0.2 ^a^	13.5 ± 0.24 ^b^	13.4 ± 0.31 ^b^***
Cyanidin (μg/day)	21.8 ± 0.17 ^c^	21.4 ± 0.23 ^c^	24.9 ± 0.27 ^a^	22.8 ± 0.35 ^b^***
DII	−19.8 ± 0.1	−20 ± 0.13	−20.1 ± 0.15	−20.2 ± 0.2
Total anthocyanins (μg/day)	24.9 ± 0.2 ^c^	24.8 ± 0.28 ^c^	43.1 ± 0.28 ^a^	41.0 ± 0.40 ^b^***^+++###^
Flavonoids (μg/day)	8.54 ± 0.05 ^c^	8.82 ± 0.07 ^a^	14.6 ± 0.08 ^a^	14.7 ± 0.11 ^a^***^+^
Isoflavonoids (μg/day)	6.70 ± 0.04 ^b^	6.88 ± 0.07 ^b^	11.6 ± 0.07 ^a^	11.5 ± 0.09 ^b^***

Values represent adjusted means and standard errors after adjusting for covariates of age, gender, energy intake, residence area, education, smoking, alcohol intake, and physical activity. An independent variable was eliminated from the covariates. L-EN-Lean, energy intake lower than estimated energy requirement and lean; L-EN-Obe, low energy intake and obese; H-EN-Lean, high energy intake and lean; H-EN-Obe, high energy intake and obese. EER, estimated energy requirement; DII, dietary inflammatory index; flavonoids = quercetin + luteolin+ kaempferol + apigenin; total anthocyanins = cyanidin + delphinidin + malvidin + pelargonidin + peonidin + petunidin; isoflavonoids = genistein + daidzein + genistein. ^++^ Significant difference with obesity by two-way ANOVA at *p* < 0.01 and ^+++^ at *p* < 0.01. * Significant difference with energy intake by two-way ANOVA at *p* < 0.05 and *** at *p* < 0.001 . ^#^ Significant interaction between energy intake and obesity by two-way ANOVA at *p* < 0.05 and ^###^ at *p* < 0.001. ^a–d^ Different letters indicated significant differences among the groups by Tukey test at *p* < 0.05. ^$$$^ Significantly different among four groups by chi-square test at *p* < 0.05.

**Table 4:** Characteristics of the ten genetic variants used for the generalized multifactor dimensionality reduction analysis.

CHR	SNP	Gene Names	BP	A1	A2	OR	SE	*p*	MAF	HWE_P	Location
1	rs506589	*SEC16B*	177894287	C	T	1.131	0.01786	5.56 × 10^−12^	0.2845	0.5099	Intron
2	rs1965122	*ADCY3*	25052450	T	C	1.085	0.01634	3.65 × 10^−7^	0.4477	0.5591	Intron
3	rs73078824	*PBRM1*	52624387	T	G	0.9055	0.01989	4.07 × 10^−7^	0.2188	0.3341	Nmd transcript
11	rs925947	*BDNF-AS*	27667367	T	G	0.9128	0.01637	2.47 × 10^−8^	0.457	0.2032	Intron
11	rs6265	*BDNF*	27679916	T	C	0.9121	0.01637	1.87 × 10^−8^	0.4588	0.1481	Missense
16	rs1421085	*FTO*	53800954	C	T	1.166	0.02412	2.07 × 10^−10^	0.1245	0.4604	Nmd transcript
19	rs1444988703	*GIPR*	46175046	A	T	1.109	0.01649	3.76 × 10^−10^	0.4066	0.4414	Nmd transcript
19	rs9636135	*QPCTL*	46200776	T	C	0.9195	0.01653	3.79 × 10^−7^	0.4197	0.5474	Nmd transcript
19	rs10408067	*SYMPK*	46363536	G	A	0.9216	0.0165	4.56 × 10^−7^	0.4193	0.3214	Intron
19	rs796090051	*DPRX*	54111540	A	C	0.9015	0.02041	3.74 × 10^−7^	0.2042	0.585	Intron

CHR, chromosome; SNP, single-nucleotide polymorphism; BP, position of SNP in chromosome. A1, minor allele; A2, major allele; MAF, minor allele frequency; HWE_P, *p* value for Hardy–Weinberg equilibrium. OR, odds ratios for serum LDL concentration in the reference of the major allele; *p*, *p* value for OR adjusted for residence area, gender, age, energy intake, alcohol intake, activity, smoke, education, and medication for metabolic diseases.

**Table 5:** (**A**) Body composition and metabolic syndrome (MetS) and its components according to the PRS with 9 genetic variants linked to obesity risk in low energy intake. (**B**) Body composition and metabolic syndrome (MetS) and its components according to the brain-derived neurotrophic factor (*BDNF*) rs6265 genetic variants linked to obesity risk in low energy intake.

(**A**)			
	**Low PRS (n = 13,638)**	**Medium PRS (n = 17,698)**	**High PRS (n = 4876)**
BMI (kg/m^2^)	23.6 ± 0.02 ^c^	24 ± 0.02 ^b^	24.3 ± 0.04 ^a^***
Skeletal muscle mass index (kg/m^2^)	7.03 ± 0.005 ^a^	7 ± 0.004 ^b^	6.96 ± 0.009 ^c^***
Body fat (%)	27.3 ± 0.03 ^c^	27.7 ± 0.03 ^b^	28.1 ± 0.05 ^a^***
Waist circumferences (cm)	80.6 ± 0.07 ^c^	81.3 ± 0.06 ^b^	82 ± 0.11 ^a^***
Hip circumferences (cm)	93.8 ± 0.05 ^c^	94.2 ± 0.04 ^b^	94.7 ± 0.08 ^a^***
WHR	0.859 ± 0.0005 ^c^	0.863 ± 0.0004 ^b^	0.865 ± 0.0008 ^a^***
Serum triglyceride (mg/dL)	127.1 ± 0.76	128.1 ± 0.67	129.7 ± 1.28
Serum glucose (mg/dL)	95.8 ± 0.18	95.8 ± 0.16	96.3 ± 0.3
Serum HDL (mg/dL)	53.2 ± 0.11	53 ± 0.1	53.1 ± 0.18
SBP (mmHg)	122.5 ± 0.12 ^b^	123 ± 0.11 ^a^	123.4 ± 0.21 ^a^**
DBP (mmHg)	75.9 ± 0.08 b	76.2 ± 0.07 ^a^	76.4 ± 0.14 ^a^**
MetS (Yes, N, %)	1822 (13.4)	2622 (14.8)	774 (15.9) ***
(**B**)			
	**Risk Allele (n = 10,579)**	**Heterozygotes (n = 18,066)**	**Non-Risk Allele (n = 7567)**
Body mass index (kg/m^2^)	24.1 ± 0.03 ^a^	23.9 ± 0.02 ^b^	23.7 ± 0.03 ^c^***
Skeletal muscle mass index (kg/m^2^)	6.99 ± 0.006 ^b^	7 ± 0.004 ^ab^	7.02 ± 0.007 ^a^*
Body fat (%)	27.8 ± 0.04 ^a^	27.6 ± 0.03 ^b^	27.4 ± 0.04 ^c^***
Waist circumferences (cm)	81.4 ± 0.08 ^a^	81.1 ± 0.06 ^b^	80.8 ± 0.09 ^c^***
Hip circumferences (cm)	94.3 ± 0.06 ^a^	94.1 ± 0.04 ^b^	93.9 ± 0.07 ^c^***
WHR	0.86 ± 0.001	0.86 ± 0	0.86 ± 0.001
Serum triglyceride (mg/dL)	128 ± 0.87	128 ± 0.66	127.7 ± 1.03
Serum glucose (mg/dL)	96.1 ± 0.2	95.7 ± 0.16	95.7 ± 0.24
Serum HDL (mg/dL)	53 ± 0.12	53.1 ± 0.1	53.1 ± 0.15
Serum LDL (mg/dL)	118.9 ± 0.27	118.9 ± 0.20	118.5 ± 0.32
SBP (mmHg)	122.9 ± 0.14	122.9 ± 0.11	122.7 ± 0.17
DBP (mmHg)	76.1 ± 0.09	76.1 ± 0.07	76 ± 0.11
MetS (Yes, N, %)	1559 (14.7)	2616 (14.5)	1043 (13.9)

Values represent adjusted means ± standard errors. The covariates included age, gender, energy intake, residence area, education, smoking, alcohol intake, fat intake, physical activity, and any medication for metabolic diseases. An independent variable was eliminated from the covariates. PRS, polygenic risk score; WHR, ratio of waist circumference and hip circumference; HDL, high-density lipoprotein; SBP, systolic blood pressure; DBP, diastolic blood pressure; Low-PRS 0–8, Medium-PRS 9–12 high-PRS ≥ 13. * Significant difference among the PRS in A or *BDNF* rs6265 alleles in B by one-way ANOVA at *p* < 0.05, ** at *p* < 0.01, and *** at *p* < 0.001. ^a–c^ Different letters indicated significant differences among the groups by Tukey test at *p* < 0.05.

**Table 6:** Bioactive compounds to lower binding energy with BDNF Val66Met WT and MT protein.

Name	WT_BE (kcal/mol)	MT_BE (kcal/mol)	Type for Low BE	Cluster by UMP	Food Source
Valolaginic acid	−11.6	−11.6	Common	C−3	guava
Torvoside C	−11.4	−10.3	Common	C−3	fruits
Casuariin	−11.3	−11.3	Common	C−3	cloves, pomegranate
Pipercyclobutanamide B	−11.3	−11.3	Common	C−3	herbs, spices
Putranjivain A	−11.1	−11	Common	C−0	fruits
Trisjuglone	−10.7	−10.7	Common	C−0	common walnut, nuts
Casuarinin	−10.7	−10.7	Common	C−0	Malabar plum, feijoa, herbs, spices, pomegranate
Stachyurin	−10.7	−10.7	Common	C−0	guava
Asterlingulatoside D	−10.7	−10.6	Common	C−0	*Aster lingulatus*
Fagopyrin	−10.6	−10.3	Common	C−0	common buckwheat, cereals, cereal products
Kaempferol 3-O-rhamnosyl-rhamnosyl-glucoside	−10.1	−10.1	Common	C−1	rosemary, common thyme, capers, common sage
Torvoside D	−10.1	−10.1	Common	C−1	fruits
Punicafolin	−10.1	−10.1	Common	C−1	pomegranate
Luteolin 7-O-diglucuronide	−10	−10	Common	C−2	common verbena, lemon verbena
Ophiopogonin C′	−10	−10	Common	C−2	onion family vegetables
(3b,16a)-Dihydroxy-12-oleanen-28-oic acid 3-[glucosyl-(1->2)-arabinoside] 28-[rhamnosyl-(1->4)-glucosyl-(1->4)-glucosyl] ester	−10.9	−8.5	WT only	C-1	fruits
(Cyanidin 3-O-beta-glucoside)(kaempferol 3-O-(2-O-beta-glucosyl-beta-glucoside)-7-O-beta-glucosiduronic acid) malonate	−10.8	−8.7	WT only	C-1	chives
Pitheduloside K	−10.8	−7.8	WT only	C-1	seeds of *Pithecellobium dulce*
(Cyanidin 3-O-(3-O-acetyl-beta-glucoside) (kaempferol 3-O-(2-O-beta-glucosyl-beta-glucoside)-7-O-beta-glucosiduronic acid) malonate	−10.7	−8.4	WT only	C-1	chives
Epicatechin-(4beta->8)-epicatechin-(4beta->6)-catechin	−10.7	−9	WT only	C-1	common grape
Kaempferol 3-rhamnosyl-(1->3)-rhamnosyl-(1->6)-glucoside	−10.1	−9.6	WT only	C-0	tea
Dioscin	−10	−9	WT only	C-0	fenugreek, yam
Cyanidin 3-(3-glucosyl-6-malonyl glucoside) 4′-glucoside	−10	−9.4	WT only	C-0	garden onion
beta1-Chaconine	−10	−9	WT only	C-0	alcoholic beverages, potato
Hesperidin	−10	−9.6	WT only	C-0	citrus fruits, Mentha longifolia
alpha-Viniferin	−9.9	−11.6	MT only	C-1	alcoholic beverages, common grape
Quillaic acid 3-[galactosyl-(1->2)-[rhamnosyl-(1->3)]-glucuronide] 28-[6-acetyl-glucosyl-(1->3)-[xylosyl-(1->4)-rhamnosyl-(1->2)]-4-acetyl-fucosyl] ester	−8.5	−10.7	MT only	C-1	bark of the *Chilean indigenous* tree (*Quillaja saponaria*)
Tragopogonsaponin Q	−8.8	−10.7	MT only	C-1	green vegetables
Ceposide D	−9.5	−10.6	MT only	C-1	garden onion, onion-family vegetables
Eleutheroside M	−8.7	−10.6	MT only	C-1	tea
Cinnamtannin A2	−8.7	−10.6	MT only	C-1	cocoa bean, Chinese cinnamon, chocolate
Chakaflavonoside A	−8.9	−10.4	MT only	C-1	tea
Epicatechin-(4beta->8)-epicatechin 3′-gallate	−9.6	−10	MT only	C-0	common buckwheat, common grape, tea
Epicatechin-(2alpha->7,4alpha->8)-epicatechin 3-galactoside	−9.5	−10	MT only	C-0	cocoa bean
Acutoside G	−9.6	−10	MT only	C-0	green vegetables

BE, binding energy with BDNF protein; WT, wild type (Val66); MT, mutated type (66Met); UMP, uniform manifold approximation and projection.

## Data Availability

All data supporting this manuscript are provided in the Supplementary Materials File.

## Supplementary Materials

The following supporting information can be downloaded at https://www.mdpi.com/article/10.3390/antiox14020170/s1, Table S1. Generalized multifactor dimensionality reduction (GMDR) of genetic variant–genetic variant interaction of genes related to obesity in the persons with low-energy-intake. Figure S1. Genetic variant distribution for obesity risk in the persons with low-energy-intake determined by genome-wide association study. (A) Manhattan plot of the *p*-value of genetic variants. The red dotted line indicates the *p*-value of the cutoff of genetic variants for obesity in low-energy-intake groups. (B) Q–Q plot of observed and expected *p*-values. The red dotted line indicates the calculated observed and expected *p*-value. It suggests a perfect match between observed and expected *p*-values.

## References

[1] Wen X., Zhang B., Wu B., Xiao H., Li Z., Li R., Xu X., Li T. (2022). Signaling pathways in obesity: Mechanisms and therapeutic interventions. Signal Transduct. Target. Ther..

[2] Hall K.D., Farooqi I.S., Friedman J.M., Klein S., Loos R.J.F., Mangelsdorf D.J., O’Rahilly S., Ravussin E., Redman L.M., Ryan D.H. (2022). The energy balance model of obesity: Beyond calories in, calories out. Am. J. Clin. Nutr..

[3] Duarte M.K.R.N., Leite-Lais L., Agnez-Lima L.F., Maciel B.L.L., Morais A.H.d.A. (2024). Obesity and Nutrigenetics Testing: New Insights. Nutrients.

[4] Bonomini F. (2023). Antioxidants and Obesity. Int. J. Mol. Sci..

[5] Manna P., Jain S.K. (2015). Obesity, Oxidative Stress, Adipose Tissue Dysfunction, and the Associated Health Risks: Causes and Therapeutic Strategies. Metab. Syndr. Relat. Disord..

[6] Wadden T.A., Tronieri J.S., Butryn M.L. (2020). Lifestyle modification approaches for the treatment of obesity in adults. Am. Psychol..

[7] Qualls-Creekmore E., Marlatt K.L., Aarts E., Bruce-Keller A., Church T.S., Clément K., Fisher J.O., Gordon-Larsen P., Morrison C.D., Raybould H.E. (2020). What Should I Eat and Why? The Environmental, Genetic, and Behavioral Determinants of Food Choice: Summary from a Pennington Scientific Symposium. Obesity.

[8] Li J., Song F. (2023). A causal relationship between antioxidants, minerals and vitamins and metabolic syndrome traits: A Mendelian randomization study. Diabetol. Metab. Syndr..

[9] Franco-San Sebastián D., Alaniz-Monreal S., Rabadán-Chávez G., Vázquez-Manjarrez N., Hernández-Ortega M., Gutiérrez-Salmeán G. (2023). Anthocyanins: Potential Therapeutic Approaches towards Obesity and Diabetes Mellitus Type 2. Molecules.

[10] Park S. (2022). Interaction of polygenic variants specific for abdominal obesity risk with energy metabolism in large Korean cohorts. Nutr. Bull..

[11] Daily J.W., Park S. (2023). Association of Plant-Based and High-Protein Diets with a Lower Obesity Risk Defined by Fat Mass in Middle-Aged and Elderly Persons with a High Genetic Risk of Obesity. Nutrients.

[12] Escalante-Aburto A., Mendoza-Córdova M.Y., Mahady G.B., Luna-Vital D.A., Gutiérrez-Uribe J.A., Chuck-Hernández C. (2023). Consumption of dietary anthocyanins and their association with a reduction in obesity biomarkers and the prevention of obesity. Trends Food Sci. Technol..

[13] Mahboob A., Samuel S.M., Mohamed A., Wani M.Y., Ghorbel S., Miled N., Büsselberg D., Chaari A. (2023). Role of flavonoids in controlling obesity: Molecular targets and mechanisms. Front. Nutr..

[14] Park S. (2023). Association of polygenic risk scores for insulin resistance risk and their interaction with a plant-based diet, especially fruits, vitamin C, and flavonoid intake, in Asian adults. Nutrition.

[15] Park S., Kim C., Wu X. (2022). Development and Validation of an Insulin Resistance Predicting Model Using a Machine-Learning Approach in a Population-Based Cohort in Korea. Diagnostics.

[16] Wu X., Park S. (2021). An Inverse Relation between Hyperglycemia and Skeletal Muscle Mass Predicted by Using a Machine Learning Approach in Middle-Aged and Older Adults in Large Cohorts. J. Clin. Med..

[17] Kim E.K., Kim J.H., Kim M.H., Ndahimana D., Yean S.E., Yoon J.S., Kim J.H., Park J., Ishikawa-Takata K. (2017). Validation of dietary reference intake equations for estimating energy requirements in Korean adults by using the doubly labeled water method. Nutr. Res. Pract..

[18] Liu M., Park S. (2024). The Role of PNPLA3_rs738409 Gene Variant, Lifestyle Factors, and Bioactive Compounds in Nonalcoholic Fatty Liver Disease: A Population-Based and Molecular Approach towards Healthy Nutrition. Nutrients.

[19] Zhou J., Kim Y.K., Li C., Park S. (2024). Natural compounds for Alzheimer’s prevention and treatment: Integrating SELFormer-based computational screening with experimental validation. Comput. Biol. Med..

[20] Kumar S., Malviya R., Sundram S. (2024). Nutritional neurology: Unraveling cellular mechanisms of natural supplements in brain health. Hum. Nutr. Metab..

[21] Rodriguez R.L., Albeck J.G., Taha A.Y., Ori-McKenney K.M., Recanzone G.H., Stradleigh T.W., Hernandez B.C., Tang F.-Y.V., Chiang E.-P.I., Cruz-Orengo L. (2017). Impact of diet-derived signaling molecules on human cognition: Exploring the food–brain axis. npj Sci. Food.

[22] Johansson A., Marroni F., Hayward C., Franklin C.S., Kirichenko A.V., Jonasson I., Hicks A.A., Vitart V., Isaacs A., Axenovich T. (2010). Linkage and genome-wide association analysis of obesity-related phenotypes: Association of weight with the MGAT1 gene. Obesity.

[23] Loos R.J.F., Yeo G.S.H. (2022). The genetics of obesity: From discovery to biology. Nat. Rev. Genet..

[24] Rupérez A.I., Gil A., Aguilera C.M. (2014). Genetics of Oxidative Stress in Obesity. Int. J. Mol. Sci..

[25] Hernández-Guerrero C., Parra-Carriedo A., Ruiz-de-Santiago D., Galicia-Castillo O., Buenrostro-Jáuregui M., Díaz-Gutiérrez C. (2018). Genetic polymorphisms of antioxidant enzymes CAT and SOD affect the outcome of clinical, biochemical, and anthropometric variables in people with obesity under a dietary intervention. Genes Nutr..

[26] Jiménez-Ortega R.F., Aparicio-Bautista D.I., Becerra-Cervera A., López-Montoya P., León-Reyes G., Flores-Morales J., Castillejos-López M., Hidalgo-Bravo A., Salmerón J., Rivera-Paredez B. (2023). Association Study between Antioxidant Nutrient Intake and Low Bone Mineral Density with Oxidative Stress-Single Nucleotide Variants: GPX1 (rs1050450 and rs17650792), SOD2 (rs4880) and CAT (rs769217) in Mexican Women. Antioxidants.

[27] Rahal D., Chiang J.J., Huynh V.W., Bower J.E., McCreath H., Fuligni A.J. (2023). Low subjective social status is associated with daily selection of fewer healthy foods and more high-fat/high sugar foods. Appetite.

[28] Feraco A., Gorini S., Camajani E., Filardi T., Karav S., Cava E., Strollo R., Padua E., Caprio M., Armani A. (2024). Gender differences in dietary patterns and physical activity: An insight with principal component analysis (PCA). J. Transl. Med..

[29] Lundsgaard A.M., Kiens B. (2014). Gender differences in skeletal muscle substrate metabolism-molecular mechanisms and insulin sensitivity. Front. Endocrinol..

[30] Speakman J.R. (2015). The ‘Fat Mass and Obesity-Related’ (FTO) gene: Mechanisms of Impact on Obesity and Energy Balance. Curr. Obes. Rep..

[31] Wang P., Loh K.H., Wu M., Morgan D.A., Schneeberger M., Yu X., Chi J., Kosse C., Kim D., Rahmouni K. (2020). A leptin-BDNF pathway regulating sympathetic innervation of adipose tissue. Nature.

[32] Jonsson A., Renström F., Lyssenko V., Brito E.C., Isomaa B., Berglund G., Nilsson P.M., Groop L., Franks P.W. (2009). Assessing the effect of interaction between an FTO variant (rs9939609) and physical activity on obesity in 15,925 Swedish and 2511 Finnish adults. Diabetologia.

[33] Xie X., Houtz J., Liao G.Y., Chen Y., Xu B. (2023). Genetic Val66Met BDNF Variant Increases Hyperphagia on Fat-rich Diets in Mice. Endocrinology.

[34] Shi R., Lu W., Tian Y., Wang B. (2023). Intestinal SEC16B modulates obesity by regulating chylomicron metabolism. Mol. Metab..

[35] Kim M.S., Shim I., Fahed A.C., Do R., Park W.Y., Natarajan P., Khera A.V., Won H.H. (2024). Association of genetic risk, lifestyle, and their interaction with obesity and obesity-related morbidities. Cell Metab..

[36] Motsinger-Reif A.A., Reif D.M., Akhtari F.S., House J.S., Campbell C.R., Messier K.P., Fargo D.C., Bowen T.A., Nadadur S.S., Schmitt C.P. (2024). Gene-environment interactions within a precision environmental health framework. Cell Genom..

[37] Emami Kazemabad M.J., Asgari Toni S., Tizro N., Dadkhah P.A., Amani H., Akhavan Rezayat S., Sheikh Z., Mohammadi M., Alijanzadeh D., Alimohammadi F. (2022). Pharmacotherapeutic potential of pomegranate in age-related neurological disorders. Front. Aging Neurosci..

[38] Gao A.X., Xia T.C., Lin L.S., Dong T.T., Tsim K.W. (2023). The neurotrophic activities of brain-derived neurotrophic factor are potentiated by binding with apigenin, a common flavone in vegetables, in stimulating the receptor signaling. CNS Neurosci. Ther..

[39] Martínez-Coria H., Serrano-García N., López-Valdés H.E., López-Chávez G.S., Rivera-Alvarez J., Romero-Hernández Á., Valverde F.F., Orozco-Ibarra M., Torres-Ramos M.A. (2024). Morin improves learning and memory in healthy adult mice. Brain Behav..

[40] Subash S., Essa M.M., Al-Adawi S., Memon M.A., Manivasagam T., Akbar M. (2014). Neuroprotective effects of berry fruits on neurodegenerative diseases. Neural Regen. Res..

[41] Marosi K., Mattson M.P. (2014). BDNF mediates adaptive brain and body responses to energetic challenges. Trends Endocrinol. Metab..

[42] Sharebiani H., Mokaram M., Mirghani M., Fazeli B., Stanek A. (2024). The Effects of Antioxidant Supplementation on the Pathologic Mechanisms of Metabolic Syndrome and Cardiovascular Disease Development. Nutrients.

[43] Farhangi M.A. (2020). Dietary total antioxidant capacity significantly interacts with 6-P21 rs2010963 gene polymorphisms in terms of cardio-metabolic risk factors in patients with metabolic syndrome. BMC Res. Notes.

[44] Nguyen V.T., Hill B., Sims N., Heck A., Negron M., Lusk C., Galindo C.L. (2023). Brain-derived neurotrophic factor rs6265 (Val66Met) single nucleotide polymorphism as a master modifier of human pathophysiology. Neural Regen. Res..

[45] Pandit M., Behl T., Sachdeva M., Arora S. (2020). Role of brain-derived neurotropic factor in obesity. Obes. Med..

[46] Miksza U., Adamska-Patruno E., Bauer W., Fiedorczuk J., Czajkowski P., Moroz M., Drygalski K., Ustymowicz A., Tomkiewicz E., Gorska M. (2023). Obesity-related parameters in carriers of some BDNF genetic variants may depend on daily dietary macronutrients intake. Sci. Rep..

[47] Khutami C., Sumiwi S.A., Khairul Ikram N.K., Muchtaridi M. (2022). The Effects of Antioxidants from Natural Products on Obesity, Dyslipidemia, Diabetes and Their Molecular Signaling Mechanism. Int. J. Mol. Sci..

[48] Mangge H., Ciardi C., Becker K., Strasser B., Fuchs D., Gostner J.M. (2017). Influence of Antioxidants on Leptin Metabolism and its Role in the Pathogenesis of Obesity. Adv. Exp. Med. Biol..

