# Supplementary material for: Energy Intake-Dependent Genetic Associations with Obesity Risk: BDNF Val66Met Polymorphism and Interactions with Dietary Bioactive Compounds
Source: Antioxidants (Basel). 2025 Jan 30;14(2):170. doi: 10.3390/antiox14020170 (PMC11851519; doi:10.3390/antiox14020170)
Supplement: Supplementary file 1 [file antioxidants-14-00170-s001.zip › antioxidants-3419512-supplementary.pdf]

Table S1. Generalized multifactor dimensionality reduction (GMDR) of genetic variant-genetic variant interaction of genes related to obesity in low energy intake.

Model1	TRBA	TEBA	P value	CVC	TRBA	TEBA	P value	CVC
SEC16B_rs506589	0.5187	0.5188	0.001	10	0.5178	0.5179	0.001	10
Model 1 plus FTO_rs1421085	0.5241	0.5203	0.001	8	0.523	0.5183	0.001	6
Model 1 plus PBRM1_rs73078824, GIPR_rs1444988703	0.5286	0.5196	0.001	4	0.5279	0.5178	0.001	4
Model 1 plus ADCY3_rs1965122, PBRM1_rs73078824, BDNF-AS_rs925947	0.534	0.5184	0.001	3	0.5333	0.518	0.001	4
Model 1 plus ADCY3_rs1965122, BDNF_rs6265 FTO_rs1421085, GIPR_rs1444988703	0.5444	0.5265	0.001	8	0.5436	0.5271	0.001	8
Model 4 plus FTO_rs1421085, GIPR_rs1444988703	0.5571	0.5197	0.001	8	0.5561	0.518	0.001	6
Model 4 plus QPCTL_rs9636135, SYMPK_rs10408067, DPRX_rs796090051	0.5786	0.5114	0.0547	8	0.5777	0.5122	0.001	9
Model 7 plus FTO_rs1421085	0.6068	0.5102	0.0107	9	0.6057	0.5108	0.001	9
Model 8 plus GIPR_rs1444988703,	0.6401	0.518	0.001	10	0.6385	0.5159	0.001	10
Model 9 plus BDNF-AS_rs925947	0.6544	0.509	0.001	10	0.6527	0.51	0.001	10

Fig. S1A

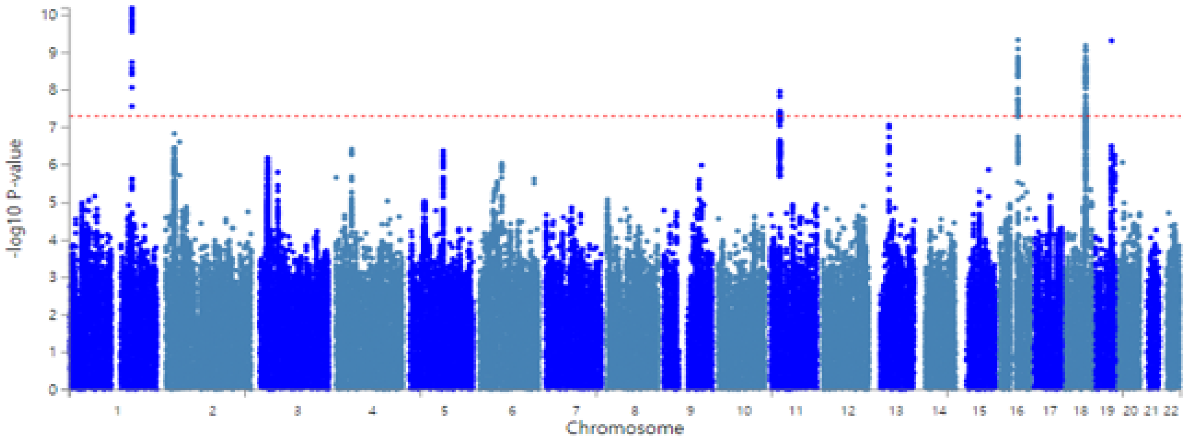

Fig. S1B

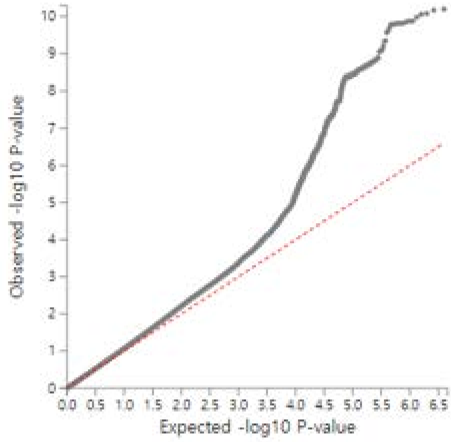

Fig. S1C

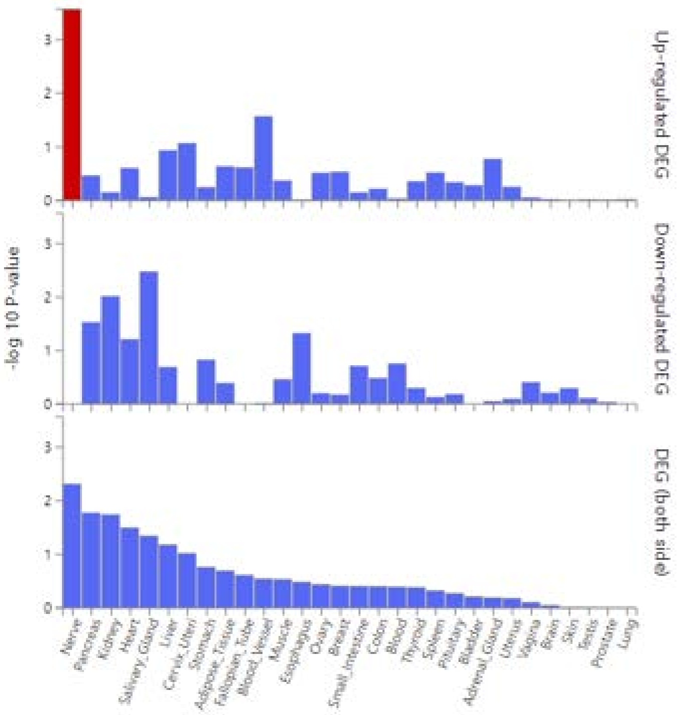

Figure S1. Genetic variant distribution for obesity risk in low energy intake by a genome-wide association study.

(A) Manhattan plot of the p-value of genetic variants. The red dotted line indicates the p-value of the cutoff of genetic variants for the obesity in low energy intake (B) Q–Q plot of observed and expected p-values. The red dotted line indicated the calculated observed and expected p-

value. It indicates the perfect matching between observed and expected p-values.
